# Effect of Exosome-loaded hsa-miR-203a-3p on Triple Negative Breast Cancer Cell Viability, Migration, and Gene Targets: In Silico and In Vitro Study

**DOI:** 10.34172/apb.45269

**Published:** 2026-05-23

**Authors:** Syamsul Arif Ardiansyah, Habibullah Noficandra, Pamungkas Bagus Satriyo, Dwi Aris Agung Nugrahaningsih, Sofia Mubarika Haryana

**Affiliations:** ^1^Biotechnology Master Program, Postgraduated school, Universitas Gadjah Mada, Indonesia; ^2^Department of Pharmacology, Faculty of Medicine, Public Health, and Nursing, Universitas Gadjah Mada, Yogyakarta, Indonesia; ^3^Department of Histology and Cell Biology, Faculty of Medicine, Public Health, and Nursing, Universitas Gadjah Mada, Yogyakarta, Indonesia

**Keywords:** Triple negative breast cancer, Exosome, hsa-miR-203a-3p, In silico

## Abstract

**Purpose:**

Triple-negative breast cancer (TNBC) is an aggressive breast cancer subtype lacking targeted therapy options. MicroRNA-based therapy, particularly hsa-miR-203a-3p, has shown tumor-suppressive potential; however, an effective delivery system is required to enhance its stability and cellular uptake. Exosomes represent a promising natural carrier for miRNA delivery.

**Methods:**

Exosomes derived from UC-MSCS secretome were isolated and characterized using Nanoparticle Tracking Analysis (NTA). Exosome-loaded hsa-miR-203a-3p (ExomiR) was generated using the Exo-Fect transfection system. Functional assays, including MTT and wound-healing assays, were performed on TNBC Hs578T cells. qRT-PCR was used to measure miRNA uptake. In silico analysis utilizing TargetScan, TarBase, DAVID, and the TNBC dataset GSE65194 was performed to identify target genes and pathways.

**Results:**

ExomiR significantly reduced TNBC cell viability in a dose-dependent manner, with viability dropping below 50% at the 2×dose. Migration assays showed the strongest inhibition at 1×dose at 12 hours (37.4±13%, *P*<0.01) and at 2×dose at 24 hours (32.3±6.18%, *P*<0.0001). ExomiR treatment yielded an 87-fold increase in intracellular hsa-miR-203a-3p expression. In silico analysis identified 23 upregulated TNBC-related target genes, including CCNB1 and CDK1, which regulate cell cycle, p53 signaling, and cellular senescence pathways.

**Conclusion:**

Exosome-mediated delivery enhances hsa-miR-203a-3p uptake and effectively suppresses cell viability and migration in TNBC cells. Computational analysis further indicates that hsa-miR-203a-3p regulates key oncogenic pathways, supporting its potential as a therapeutic agent.

## Introduction

 Breast cancer remains the most prevalent malignancy among women worldwide and is the leading cause of cancer-related mortality. In 2020 alone, breast cancer was responsible for approximately 685,000 deaths globally, with a significant burden in Indonesia, where 22,000 fatalities were reported.^1^ This high mortality rate underscores the urgent need for more effective diagnostic and therapeutic strategies. Breast cancer is a heterogeneous disease, classified into several molecular subtypes based on the expression of estrogen receptor (ER), progesterone receptor (PR), and human epidermal growth factor receptor 2 (HER2). The triple-negative breast cancer (TNBC) subtype, characterized by the absence of these three receptors, is considered the most aggressive and difficult to treat, primarily due to the lack of targeted hormonal or HER2-directed therapies.^2^

 Among all breast cancer subtypes, TNBC is associated with higher recurrence rates, increased metastatic potential, and poorer survival outcomes. Conventional treatment options for TNBC remain largely limited to surgery, radiation therapy, and chemotherapy, which often result in significant toxicity and therapeutic resistance. The rapid progression and poor prognosis of TNBC highlight the need for novel therapeutic approaches that target key molecular regulators of tumor growth, proliferation, and metastasis.^3^ One promising strategy involves the use of microRNAs (miRNAs), small non-coding RNAs that play a pivotal role in gene regulation and cancer progression.^4^

 MicroRNAs have been widely studied for their ability to modulate post-transcriptional gene expression, thereby influencing crucial cellular processes such as proliferation, apoptosis, migration, and invasion. In the context of TNBC, several miRNAs have been identified as potential oncogenes or tumor suppressors, regulating pathways involved in tumor initiation and metastasis.^5^ One of the most promising tumor-suppressive miRNAs in breast cancer is hsa-miR-203a-3p, which has been reported to exhibit anti-metastatic properties. Studies have demonstrated that hsa-miR-203a-3p expression is significantly reduced in TNBC patients, with its downregulation correlating with advanced disease stages and poorer clinical outcomes. Furthermore, functional studies in TNBC cell lines have shown that the introduction of hsa-miR-203a-3p mimics leads to significant suppression of proliferation, migration, and colony formation, suggesting its potential as a therapeutic agent.^6,7^

 Despite the promising role of hsa-miR-203a-3p in TNBC, challenges remain in effectively delivering miRNA-based therapies to tumor cells while minimizing off-target effects and degradation in circulation. In this regard, exosome-based drug delivery systems have emerged as a powerful tool for the targeted delivery of therapeutic molecules. Exosomes are nano-sized extracellular vesicles that play a crucial role in intercellular communication, transporting bioactive molecules such as proteins, lipids, and RNAs between cells. Their natural lipid bilayer composition provides protection against enzymatic degradation, enhancing the stability and efficacy of miRNA-based therapies,^8^ Recent studies have highlighted the non-toxic and highly efficient nature of exosome-mediated delivery, making them an attractive platform for targeted miRNA therapy in breast cancer, including TNBC.^9,10^

 Although significant advancements have been made in miRNA-based therapeutics and exosome research, the potential of exosome-loaded hsa-miR-203a-3p for TNBC treatment remains largely unexplored. Moreover, a comprehensive understanding of hsa-miR-203a-3p’s target genes within the heterogeneous landscape of TNBC is still lacking. Unlike previous studies that evaluated exosomal miRNA delivery in general cancer models, the present study specifically investigates UC-MSCS–derived exosomes as carriers of hsa-miR-203a-3p in a TNBC-specific context. Additionally, this work integrates in vitro functional assays with TNBC-specific transcriptomic data (GSE65194), enabling identification of high-confidence gene targets relevant to TNBC biology. To our knowledge, this is the first study combining exosome-mediated delivery of hsa-miR-203a-3p with in silico pathway profiling focused exclusively on TNBC. Given these gaps, this study aims to investigate the functional effects of exosome-loaded hsa-miR-203a-3p on TNBC cell lines (Hs578T) and predict its potential target genes through in-silico analysis. By integrating experimental and computational approaches, we seek to provide novel insights into the molecular mechanisms regulated by hsa-miR-203a-3p and explore its potential as a therapeutic candidate for TNBC.

## Methods

###  Study Design and Setting

 This in vitro experimental study investigated the effect of exosome-loaded hsa-miR-203a-3p on TNBC Hs578T cells and predicted its target genes using computational analysis. The study was conducted in a controlled laboratory environment.

 Exosomes were isolated from UC-MSCS secretome and characterized using Nanoparticle Tracking Analysis (NTA). TNBC Hs578T cells were cultured and treated with exosomes for viability and migration assays. RNA isolation and qRT-PCR measured hsa-miR-203a-3p expression, while in silico tools identified potential target genes. Statistical analysis was performed to evaluate results.

###  Exosome Preparation

 Exosomes were isolated from umbilical cord mesenchymal stem cell (UC-MSCS) secretome, obtained from PT. Tristem Medika Indonesia using the Invitrogen^TM^ Total Exosome Isolation kit (Life Technologies, Carlsband, Massachusetts, USA). The secretome, stored frozen at -20°C, was thawed at 25°C-37°C before isolation. Exosome characterization involved size, concentration, and phenotype observation using Nanoparticle Tracking Analysis (NTA) performed by PT. Dermama, Indonesia. Exosome samples were diluted with vesicle-free saline solution or sterile phosphate-buffered saline (PBS) before analysis equipped with a 405 nm laser and NTA analysis software.

###  Cell Culture And Loading

 TNBC Hs578T cells were maintained in DMEM supplemented with 10% fetal bovine serum (FBS), 2% penicillin–streptomycin, and 0.5% fungizone (Gibco Inc., Grand Island, NY, USA) under standard conditions at 37 °C in a humidified atmosphere containing 5% CO₂. Upon reaching the appropriate confluence, hsa-miR-203a-3p mimics were prepared and loaded into UC-MSC–derived exosomes using the Exo-Fect^TM^ Exosome Transfection Kit (System Biosciences, Carlsbad, CA, USA) according to the manufacturer’s instructions. The synthetic hsa-miR-203a-3p mimic was obtained from Integrated DNA Technologies (IDT, USA) and corresponded to the mature miRNA sequence 5′-GUGAAAUGUUUAGGACCACUAG-3′ based on miRBase v22.1. Briefly, purified exosomes were incubated with the hsa-miR-203a-3p mimic and Exo-Fect reagent for 10 min at 37 °C, followed by purification using ExoQuick-TC precipitation buffer to remove excess reagents.

###  Cell Viability Test

 MTT assay involved eight treatments: cell control, exosome control, naked mimic-hsa-miR-203a-3p control (Naked miR), Exo-Fect exosome (EE) control (transfection without mimic miR), and ExomiR at volumes of 2x, 1x, 1/2x, and 1/4x, with 3405 cells per well in a 96-well plate. After 24-hour incubation at 37°C with 5% CO_2_, MTT reagent (0.5 mg/mL per well) was added and incubated for 4 hours. Then, 100 µL of sodium dodecyl sulfate (SDS-HCl) for lysing the cells. solution to solubilize formazan crystalssolution was added to each well and incubated overnight at room temperature. Absorbance readings were taken at 595 nm using an enzyme-linked immunosorbent assay (ELISA) reader.

###  Migration Test 

 Cells were seeded in 24-well plates and allowed to reach near confluence prior to scratching. After 24 hours of incubation at 37°C with 5% CO_2_, eight treatments were applied in triplicate. Scratch wounds were created using a 200 µL pipette tip, and images were captured at 0-, 12-, and 24-hours post-scratching using an Optilab camera connected to an inverted microscope.

###  Ribonucleic Acid (RNA) Isolation

 RNA isolation was conducted on 100,000 cells per well in a 6-well plate across treatment groups. This cell density was optimized to match the concentration of exosome-loaded hsa-miR-203a-3p administered, ensuring an appropriate treatment-to-cell ratio and sufficient RNA yield for qRT-PCR analysis. The groups are: cell control, exosome control, mimic-hsa-miR-203a-2p control (Naked miR), exo-exofect control (EE), and ExomiR at a volume of 1x. Following a 36-hour incubation period, RNA extraction was carried out using miRCURY RNA Kit/miRNeasy Tissue/Cells Kit (Qiagen NV, Hilden, Germany). The concentration of RNA eluate was assessed using nanodrop, ensuring an absorbance ratio of A260/280 between 1.8 and 2, indicating acceptable purity.

###  Quantitative Real-Time Polymerase Chain Reaction (qRT -PCR) 

 After measuring RNA concentration, complementary deoxyribonucleic acid (cDNA) synthesis was performed. RNA isolate was diluted to a concentration of 20 ng/µL using nuclease-free water. Subsequently, cDNA synthesis followed universal cDNA miRCURY LNA RT kit protocol (cat. 339340, Qiagen NV, Hilden, Germany). For qRT-PCR, miRCURY LNA SYBR Green PCR Assay kit (cat 339345, Qiagen NV, Hilden, Germany) was utilized with Primer U6 (Qiagen NV, Hilden, Germany) and Primer hsa-miR-203a-2p (Qiagen NV, Hilden, Germany) for hsa-miR-203a-3p expression. Prior to the qRT-PCR program, cDNA was further diluted at a 1:3 ratio (1 template cDNA and 3 nuclease-free water (NFW).

###  In Silico Analysis

 In silico analysis employed TargetScan (https://www.targetscan.org/) and TarBase (https://dianalab.e-ce.uth.gr/tarbasev9) datasets for hsa-miR-203a-3p target gene prediction. GSE65194 dataset identified upregulated genes in TNBC. JVenn (https://www.bioinformatics.com.cn/static/others/jvenn_en/) identified gene overlaps. Further analysis utilized David software (https://david.ncifcrf.gov/) and PrognoScan (http://dna00.bio.kyutech.ac.jp/).To enhance biological relevance, downstream analyses focused on high-confidence target genes predicted by both TargetScan and TarBase and significantly upregulated in TNBC samples from the GSE65194 dataset. Genes were further prioritized based on established functional roles in TNBC-related pathways, including cell cycle regulation, cellular senescence, and p53 signaling.

###  Statistical Analysis

 Data from each group was compared with its respective control using an independent t-test. qRT-PCR Relative miRNA expression was calculated using the ΔΔCt method, facilitating relative miRNA expression measurement between control and treatment groups. Each experiment was conducted in three biological replicates with three technical repeats per condition. Data are expressed as mean ± SD. Comparisons between treatment and control groups were performed using an independent t-test, with statistical significance set at *P* < 0.05.

## Results

###  Isolation and Characterization of Exosomes from UC-MSCS Secretome

Table 1 illustrates a range of particle sizes within the samples. For sample E1, D10 indicated that 10% of particles measure below 97.15 nm, while D50 and D90 revealed that 50% and 90% of particles are below 187.33 nm and 453.20 nm, respectively. This suggests that particles sized between 30–150 nm constitute less than 50% of sample E1. However, the average particle size in sample E1 is 109 nm. After blank subtraction, quantitative data in terms of particle count per mL indicate a pure concentration of 1.4 x 10^8^ particles/mL for particles sized between 30–150 nm in sample E1. As shown in Figure 1, NTA analysis demonstrates particle size distribution and phenotype of exosome particles.

**Table 1:** Characteristic of exosome samples

**Sample**	**D10 (nm)**	**D50 (nm)**	**D90 (nm)**	**Average size (nm)**	**Concentration (particles/mL)**	**Range Size**
PBS	7.43	77	187.80	97	1.1 x 106	30-150
E1	97.15	187.33	453.20	109	2.4 x 108	

Abbreviations: D10/D50/D90 = particle diameter percentiles; PBS = phosphate-buffered saline.

**Figure 1:**
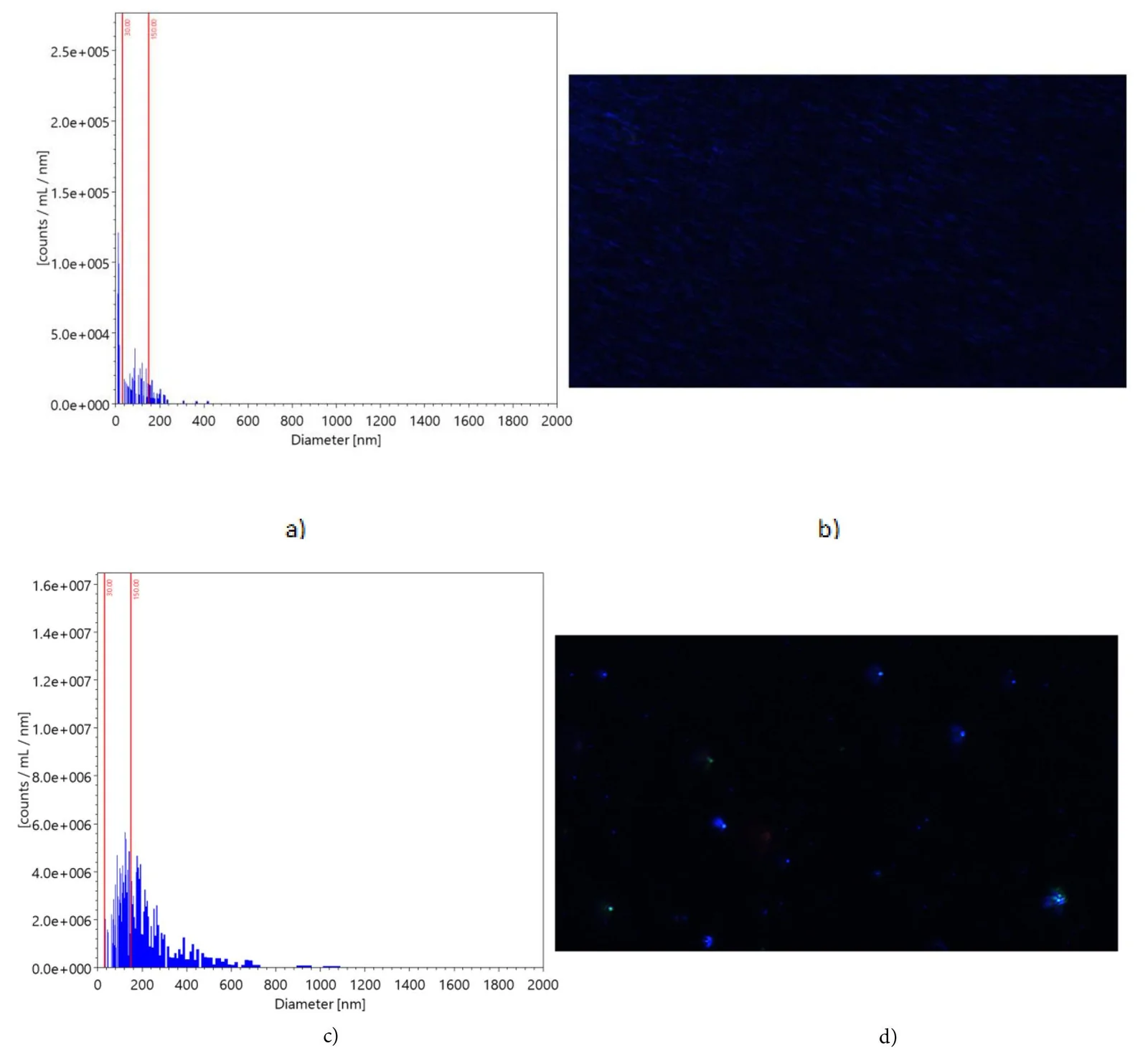
Nanoparticle Tracking Analysis (NTA) of UC-MSCS–derived exosomes. (a) Size distribution of vesicles in PBS control; (b) Phenotype of vesicles in PBS control; (c) Size distribution of UC-MSCS exosomes; (d) Phenotype of exosome particles. X-axis represents particle diameter (nm), and Y-axis represents particle concentration (particles/mL)

###  Effect of exomiR on TNBC Cell Viability

 MTT assay data indicates that exomiRs significantly reduce cell viability in a dose-dependent manner (Figure 2a). Viability decreased as exomiR volume increased: 85.1 ± 3.6% at ¼x, 7.1 ± 0.3% at ½x, 63.7 ± 1.8% at 1x, and 47 ± 1.1% at 2x. Comparing exomiR treatments with exo-exofect controls revealed significant difference (*P* < 0.01 for ¼x, *P* < 0.0001 for 1/2x, 1x, and 2x). Other controls did not differ significantly from the cell control, except for the exosome control (*P* < 0.05). Viability below 50% at the 2x dose warrants determination of the IC_50_ (Figure 2b). an inhibitory concentration equivalent to an IC_50_ value of 13.54 µL-equivalent exosome preparation. Morphological observation of Hs578T cells revealed that untreated control cells exhibited a typical spindle-shaped and elongated morphology characteristic of mesenchymal-like TNBC cells (Figure S1) In contrast, cells treated with exosome-loaded hsa-miR-203a-3p showed noticeable morphological alterations, including reduced cell spreading, increased cell rounding, and decreased cell–cell connectivity, suggesting impaired migratory behaviour (Figure S2).

**Figure 2:**
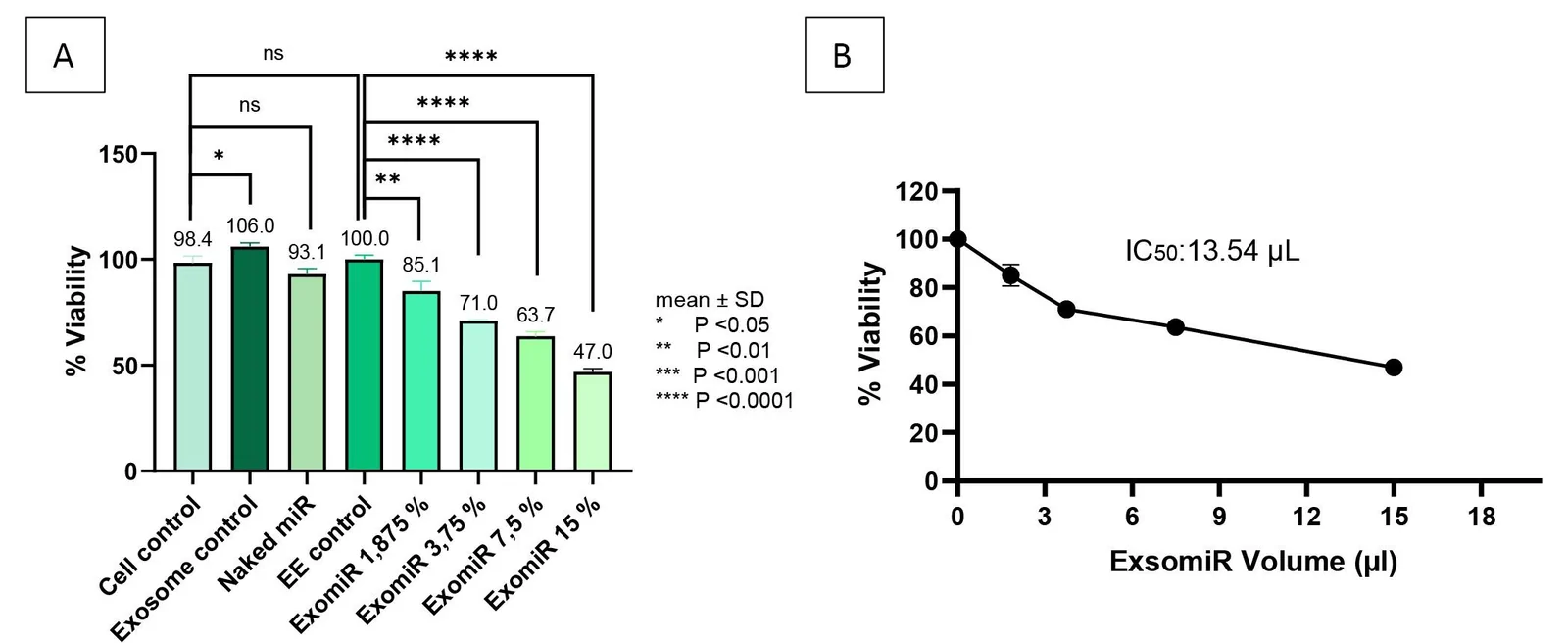
a) ExomiR effect on Hs578T cell viability: Best inhibition at 2x dose (*P* < 0.0001); b) Viability inhibition by exomiR complex on TNBC Hs578T cells across doses (2x, 1x, 1/2x, and 1/4x). IC_50_ value obtained: 13.54 µL. Error bars indicate standard deviation (SD). EE: Exo-Fect exosome control; Naked miR: mimic-hsa-miR-203a-3p without exosomal loading; Ctrl: untreated cell control

###  Effect of exomiR on TNBC Cell Migration

 Migration test revealed a significant reduction in cell migration rates with exomiR treatment compared to controls (Figure 3). When comparing migration rates between exo-exofect control and exomiR-treated groups, the most substantial inhibition at 12 hours was observed with a 1x volume, resulting in a 37.4 ± 13% reduction in migration (*P* < 0.01) (Figure 4a). At 24 hours, the highest inhibition occurred with a 2x volume, showing a 32.3 ± 6.18% reduction in migration with high statistical significance (*P* < 0.0001) (Figure 4b). No significant difference was observed in the migration rates of the control groups at both 12 and 24 hours.

**Figure 3:**
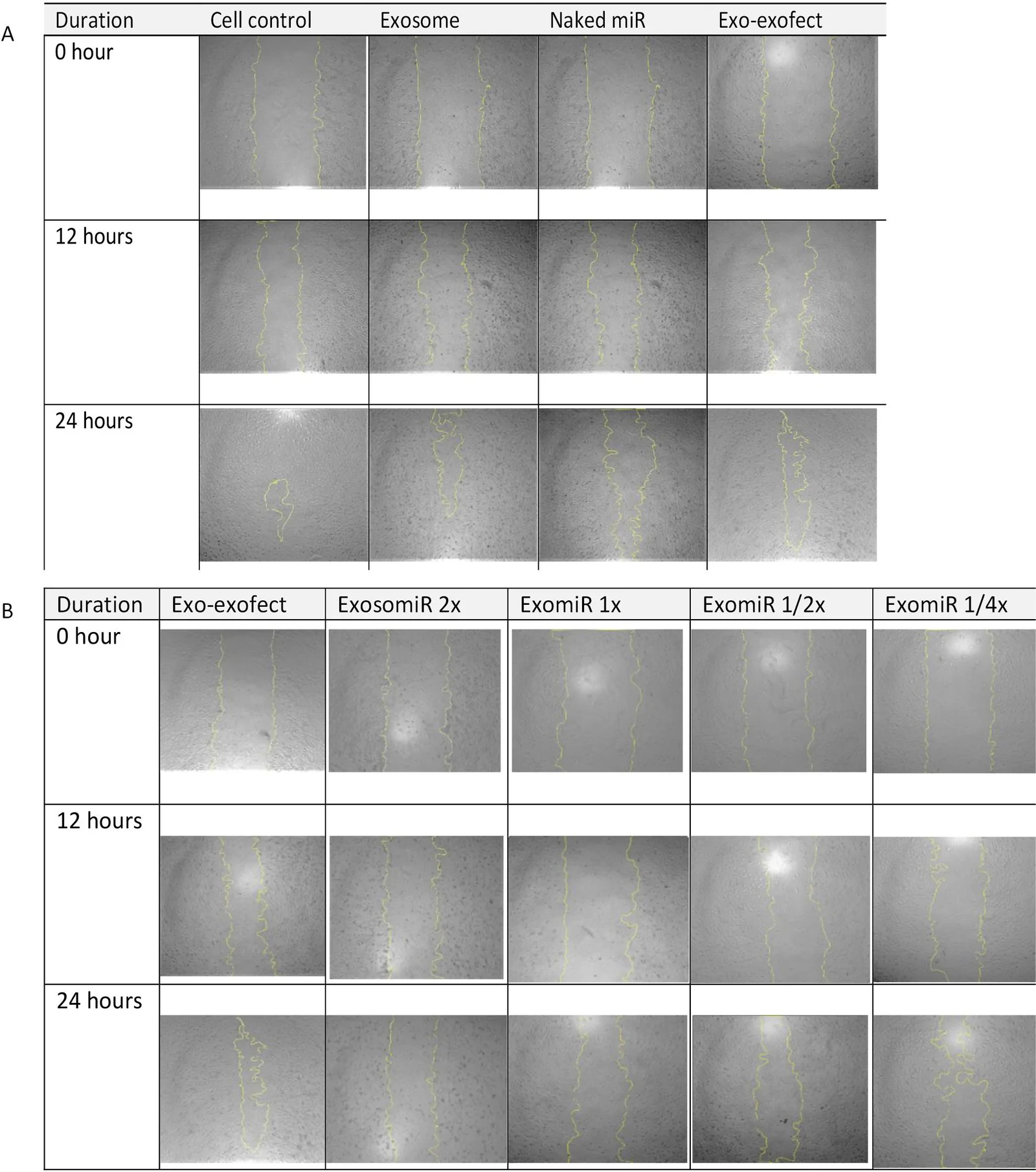
a) Observations of TNBC cell migration at 0, 12, and 24 hours between control groups. b) Observations of TNBC cell migration at 0, 12, and 24 hours between control EE and exomiR

**Figure 4:**
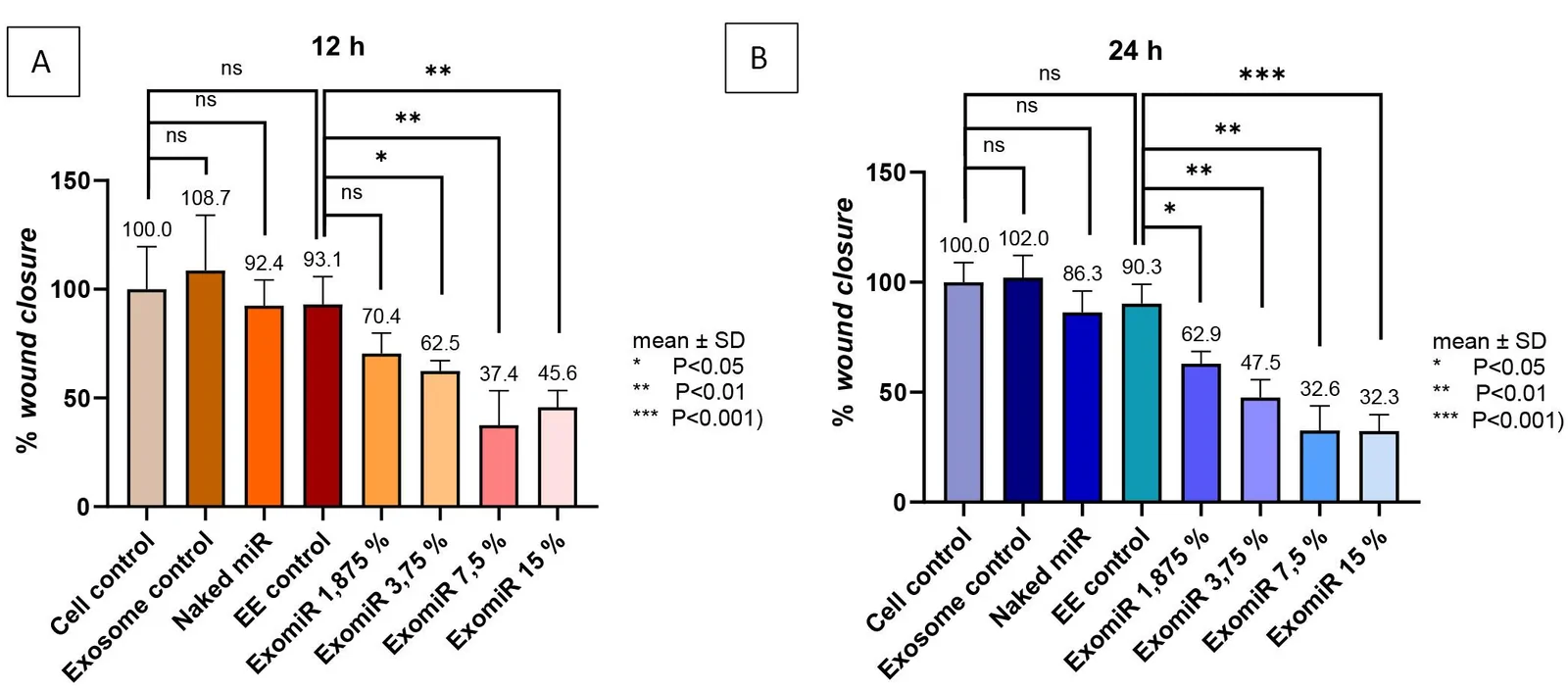
a) % Area of TNBC cells at hour 12: Best inhibition at dose 1x (*P* < 0.01); b) % Area of TNBC Hs578T cells at hour 24: Best inhibition at dose 2x (*P* < 0.001). EE: Exo-Fect exosome control; Naked miR: mimic-hsa-miR-203a-3p without exosomal loading; Ctrl: untreated cell control

###  Expression Test of hsa-miR-203a-3p in TNBC Cells Transfected with exo-miR

 MiRNA hsa-miR-203a-3p expression was assessed in TNBC cell line under different treatments compared to the control. Exo-miR treatment yielded an 87-fold increase in expression (*P* < 0.01), while mimic-hsa-miR-203a-3p treatment showed a 13-fold increase (*P* < 0.05). In contrast, EE control treatment led to a 0.68-fold increase, while exosome control treatment resulted in a 0.53-fold decrease in expression (Figure 5).

**Figure 5:**
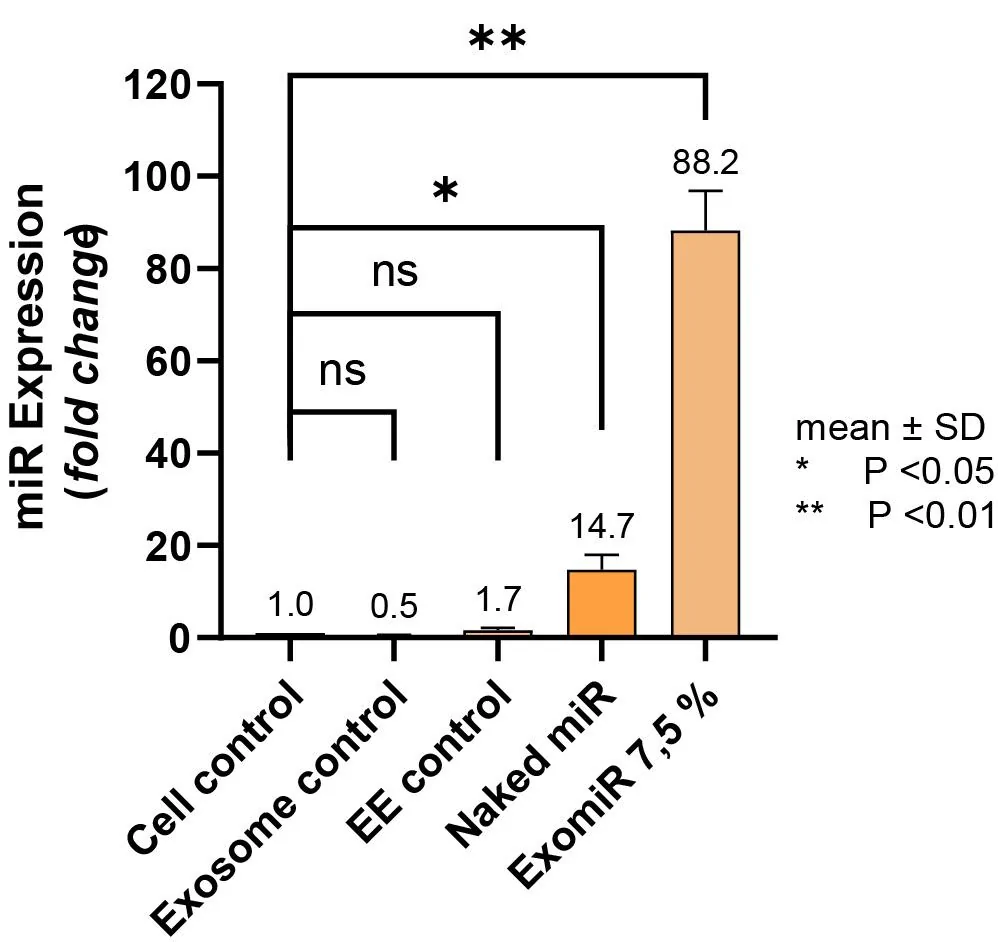
Relative expression of hsa-miR-203a-3p in Hs578T cells following different treatments. Data are presented as mean ± SD. Statistical analysis was performed using an independent t-test (*P < 0.05; **P < 0.01)

###  In Silico Analysis of Target Genes for hsa-miR-203a-3p 

 The present study methodology involved integrating target genes of hsa-miR-203a-3p from TargetScan and TarBase datasets, totaling 4640 genes, with upregulated genes in TNBC sourced from GSE65194. This dataset comprises 130 breast cancer samples and 23 technical duplicates, alongside 11 healthy breast tissue samples and 14 breast cancer cell lines. Analysis focused on comparing gene expression between TNBC and healthy breast tissue samples using GEO2R, identifying 280 genes with increased expression within a logFc range of 4 to 8-fold.

 Among the 111 genes scrutinized, 56 were identified within KEGG pathways, spanning areas such as oocyte meiosis and progesterone-mediated oocyte maturation. Additionally, 23 genes play roles in processes associated with TNBC, including cell cycle and p53 signaling pathway. PrognoScan analysis unveiled 18 genes acting as oncogenes, 3 as tumor suppressors, and 2 with ambiguous roles among the TNBC-related genes (Figure 6).

**Figure 6:**
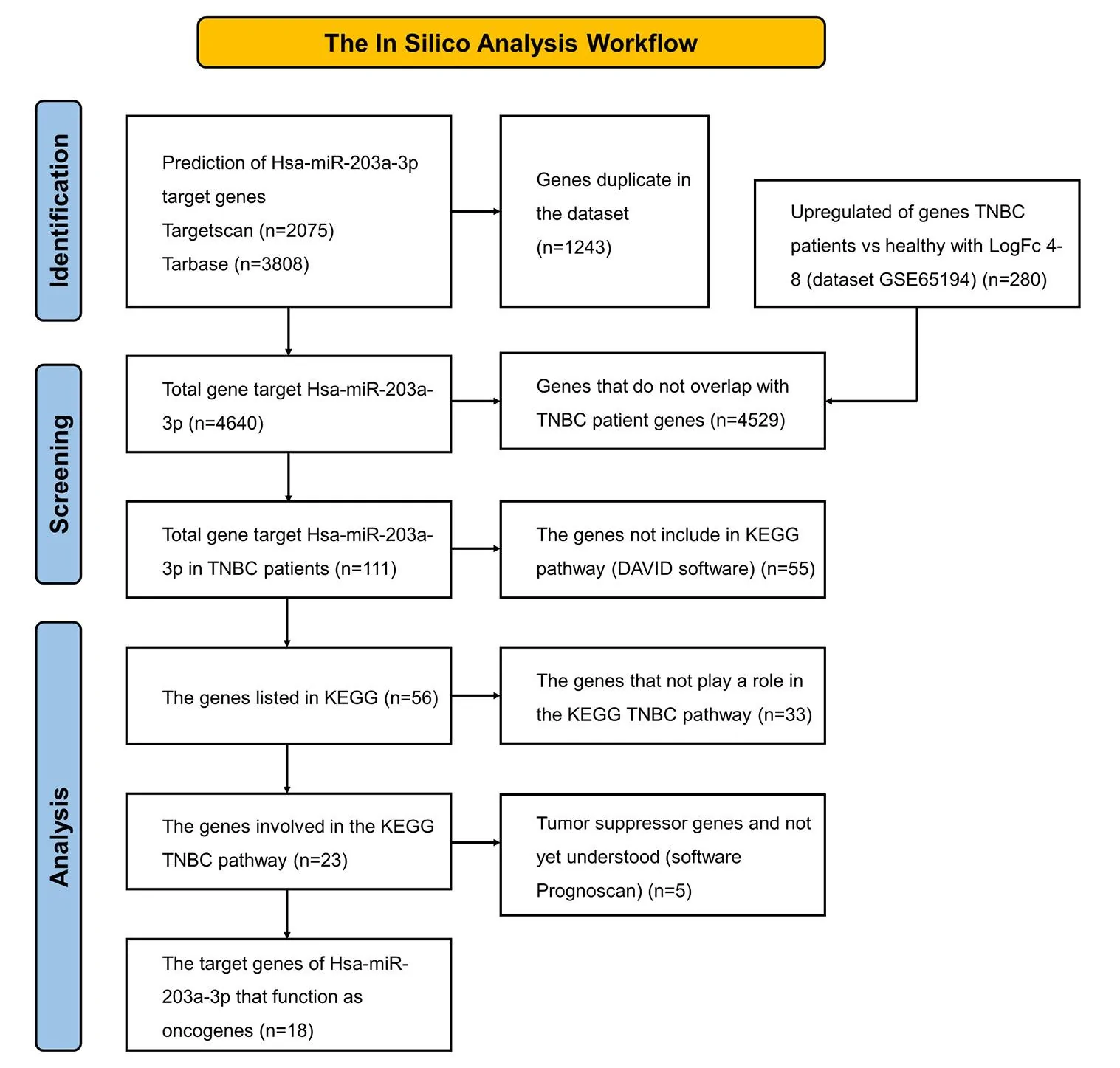
In silico analysis workflow of hsa-miR-203a-3p target genes in TNBC

 Obtained data indicates dual regulation of three pathways by two genes, CCNB1 and CDK1, involving cellular senescence, cell cycle, and the p53 signaling pathway. Additionally, three genes, CCNA2, AURKB, and RRM2, are implicated in two pathways each: CCNA2 in cellular senescence and the cell cycle, AURKB in the cell cycle and Pyrimidine metabolism, and RRM2 in p53 signaling pathway and Pyrimidine metabolism. Furthermore, MAD2L1 demonstrates substantial influence from Hsa-miR-203a-3p, with seven identified binding sites between miRNA and the gene. Gene data, logFC expression, KEGG pathways, and prognosis are integrated into Cytoscape for visualizing the interaction between hsa-miR-203a-3p and metabolic genes (Figure 7) and hsa-miR-203a-3p inhibition mechanism scheme (Figure 8).

**Figure 7:**
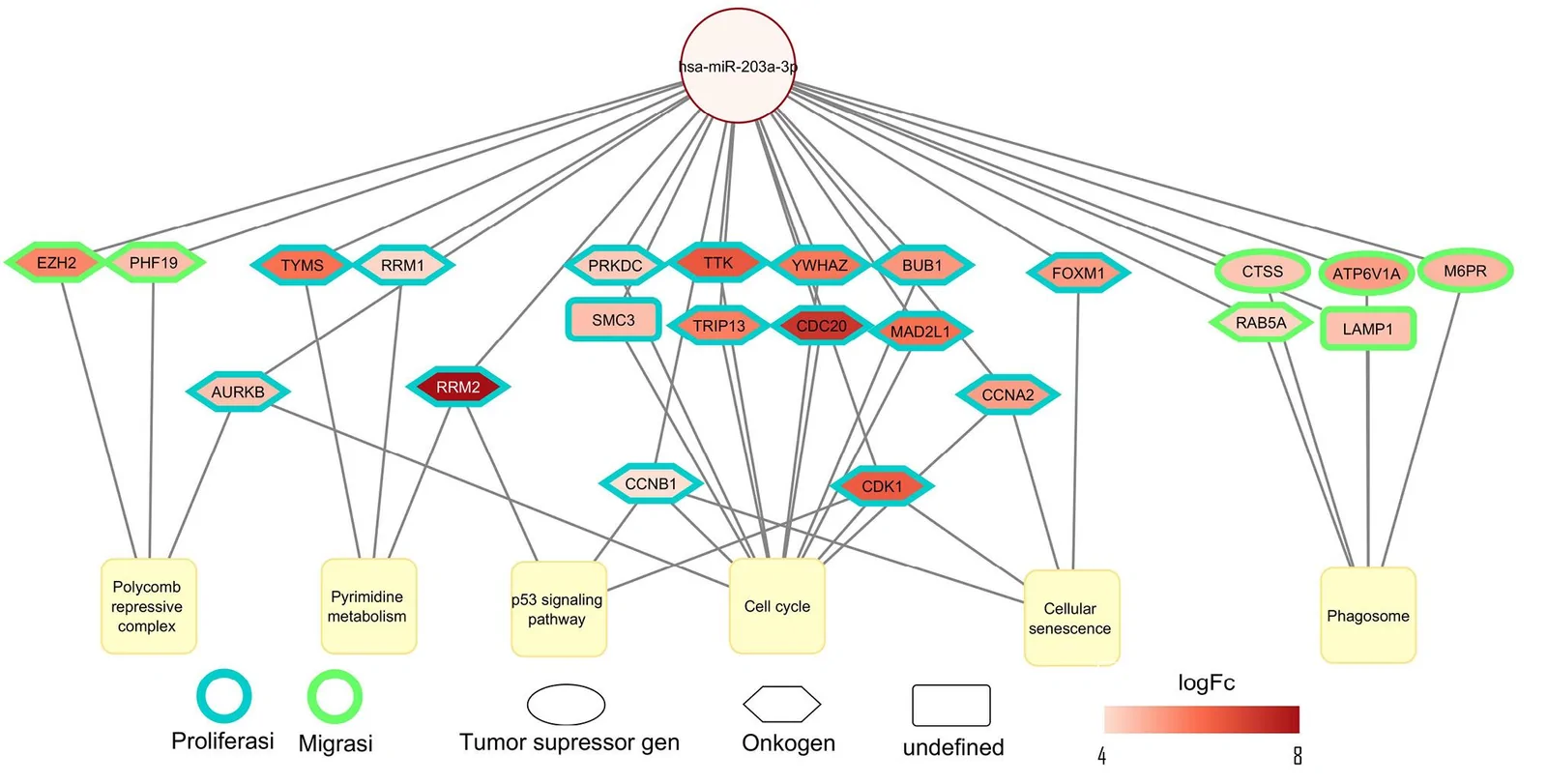
Cytoscape visualization of 23 hsa-miR-203a-3p target genes upregulated in TNBC: 18 oncogenes, 3 tumor suppressors, and 2 with unknown functions. Pathway nodes were exported from Cytoscape using high-resolution vector settings to ensure readability of gene labels and functional categories

**Figure 8:**
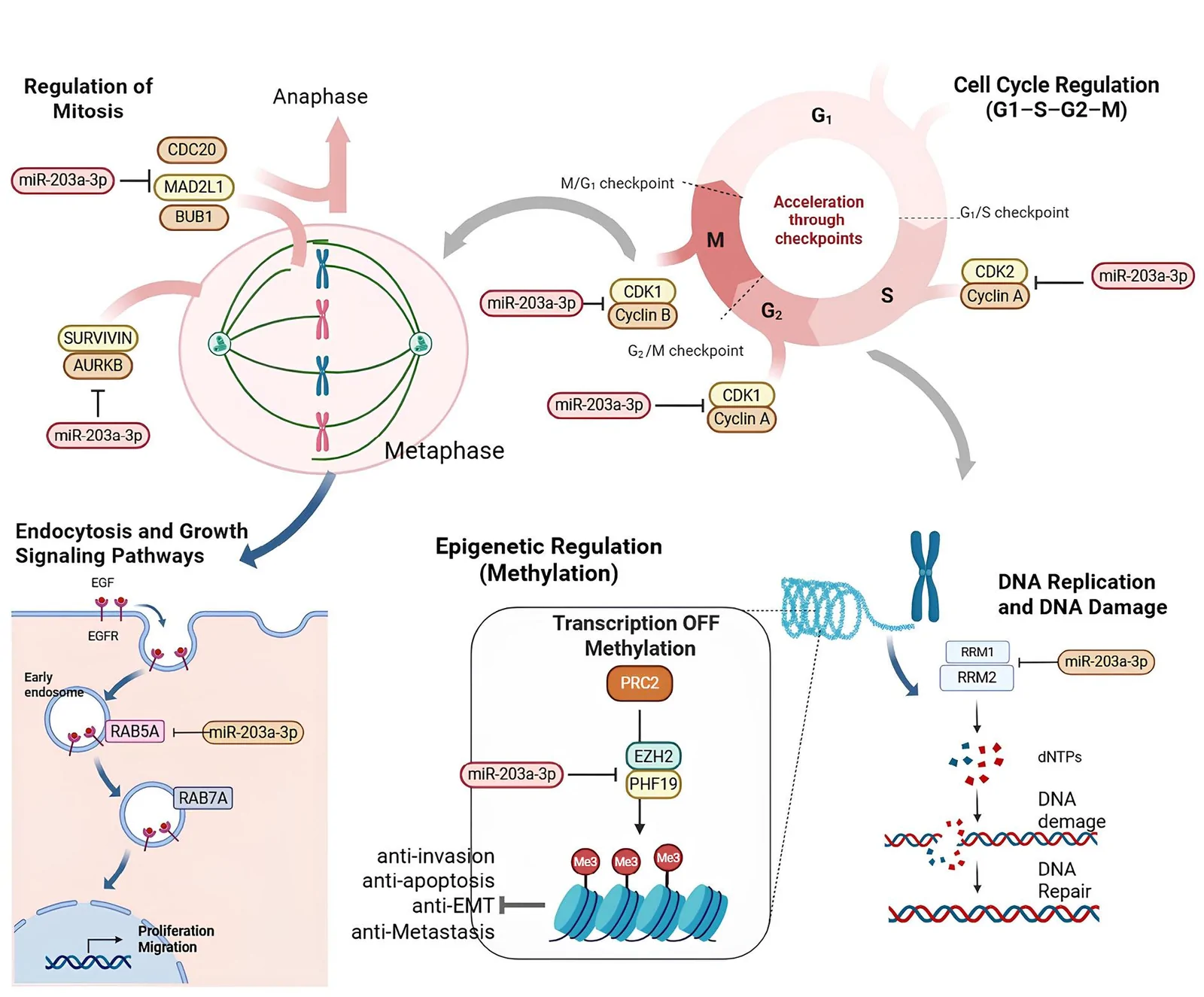
Schematic representation of the proposed mechanism through which hsa-miR-203a-3p regulates TNBC-related pathways, including cell cycle control, p53 signaling, and cellular senescence. miR-203a-3p functions as a key regulatory molecule that suppresses cancer progression through multiple coordinated molecular pathways. Its inhibitory effects begin at the level of the cell cycle, where miR-203a-3p downregulates the CDK2/Cyclin A complex during the G1/S phase, as well as CDK1/Cyclin A and CDK1/Cyclin B during the G2/M phase, thereby slowing cell cycle progression. During mitosis, miR-203a-3p further targets checkpoint proteins such as CDC20, MAD2L1, and BUB1, disrupting the transition from metaphase to anaphase and impairing proper cell division. In parallel, miR-203a-3p suppresses the expression of RRM1 and RRM2, which are essential for deoxyribonucleotide (dNTP) synthesis, leading to impaired DNA replication and increased DNA damage. At the epigenetic level, miR-203a-3p inhibits the PRC2 complex, including EZH2 and PHF19, thereby reducing histone methylation and enabling the reactivation of tumor suppressor genes. Additionally, miR-203a-3p interferes with endocytic trafficking and growth signaling by targeting RAB5A and RAB7A, while also suppressing anti-apoptotic proteins such as survivin and AURKB. Collectively, these effects promote apoptosis and inhibit cell proliferation, migration, and metastasis. Overall, miR-203a-3p acts as a tumor suppressor through a coordinated multi-target inhibitory mechanism

 From the integrated dataset, a refined subset of 23 high-confidence genes was selected for further analysis. These genes were consistently predicted as hsa-miR-203a-3p targets, significantly upregulated in TNBC samples, and functionally implicated in key oncogenic pathways. This focused approach allowed clearer interpretation of biologically relevant regulatory networks associated with TNBC progression.

## Discussion

 Exosomes utilized in the present study were sourced from the secretome of PT. Tristem Medika Indonesia, originating from UC-MSCS. UC-MSCS was chosen for their rapid proliferation, low immunogenicity, and absence of adverse effects in vivo.^11,12^ NTA characterization confirmed successful isolation, with exosome size averaging 109 nm, within the typical range of 30–150 nm.^13^

 The present study focused solely on TNBC, and the specificity of exosome-mediated miR-203a-3p delivery for this subtype remains undetermined. Since breast cancer subtypes differ markedly in molecular profiles, future studies are needed to compare responses across luminal and HER2-positive models.

 In the present study, exosome control showed a modest increase in viability, consistent with exosomes’ role as growth stimulators derived from UC-MSCS secretome. This aligns with Ellistasari *et al* demonstrating exosomes’ potential in promoting angiogenesis, repair, and cell proliferation in degenerative diseases.^14^ Neither the naked miR control nor the EE control exhibited significant difference compared to cell control in the present study. However, EE control still demonstrated higher viability, possibly due to exosomes’ angiogenic properties. In contrast, naked miR, a short non-coding RNA, is susceptible to degradation without effective delivery systems.^15^ Exosomes, with membrane receptors facilitating interaction with TNBC cell membranes, offer a promising delivery platform, unlike naked miRNA.^16^ UC-MSCS exosomes possess inherent pro-regenerative and modulatory properties, which may influence cellular behavior. Thus, part of the observed anti-proliferative or anti-migratory effects may derive from exosomal components independent of miR-203a-3p. This interaction warrants further investigation.

 MicroRNAs (miRNAs) are crucial regulators of post-transcriptional gene expression and have been extensively studied for their roles in cancer progression. In this study, we investigated the regulatory network of hsa-miR-203a-3p and its functional consequences on triple-negative breast cancer (TNBC). Previous studies have reported dual roles for miR-203a-3p, functioning either as a tumor suppressor or as a proliferation-promoting factor depending on cellular context. These discrepancies may reflect differences in tumor subtype, epigenetic regulation, or the cellular microenvironment. In TNBC, available evidence, including the present study, indicates predominantly suppressive activity. Functional annotation analysis using the Database for Annotation, Visualization, and Integrated Discovery (DAVID) revealed that 56 out of 111 target genes were enriched in key Kyoto Encyclopedia of Genes and Genomes (KEGG) pathways, including oocyte meiosis, progesterone-mediated oocyte maturation, phagosome, tuberculosis, pyrimidine metabolism, hepatitis B, Polycomb repressive complex, cell cycle, cellular senescence, and the p53 signaling pathway. Notably, 23 genes were significantly associated with TNBC progression, particularly through pathways involved in cell cycle regulation, senescence, and tumor suppressor signalling.^17^

 To assess the prognostic significance of these genes, PrognoScan, a bioinformatics platform that integrates microarray-based cancer datasets, was employed. Among the 23 genes involved in TNBC-related pathways, 18 functioned as oncogenes, 3 as tumor suppressors, while 2 exhibited undefined roles. However, a major limitation of PrognoScan is its reliance on general breast cancer datasets, lacking classification based on specific breast cancer subtypes. To address this, a literature review was conducted to validate the roles of these genes, and their interactions with hsa-miR-203a-3p were visualized using Cytoscape, providing a comprehensive network representation of their involvement in TNBC pathophysiology.^18^

###  Key Regulatory Genes Targeted by hsa-miR-203a-3p

 Among the key findings, Cyclin B1 (CCNB1) and Cyclin-Dependent Kinase 1 (CDK1) were identified as central regulators of cellular senescence, cell cycle progression, and the p53 signaling pathway. CDK1 plays a pivotal role in G2/M and G1/S transitions, facilitating proper mitotic progression and spindle formation. Dysregulation of CDK1 activity contributes to uncontrolled proliferation, a hallmark of TNBC.^19^ Similarly, CCNB1, a key mitotic regulator, interacts with CDK1 to drive the transition from G2 to mitosis. Inhibition of CCNB1/CDK1 disrupts the G2/M checkpoint, leading to cell cycle arrest and apoptosis, making them potential therapeutic targets.^20^

 Additionally, three genes were found to regulate two KEGG pathways: Cyclin A2 (CCNA2), Aurora Kinase B (AURKB), and Ribonucleotide Reductase Regulatory Subunit M2 (RRM2). CCNA2 modulates cellular senescence and cell cycle progression, interacting with Cyclin-Dependent Kinases (CDKs) to regulate DNA replication and mitotic entry.^21^ AURKB, a core component of the Chromosomal Passenger Complex (CPC), facilitates chromosome condensation, segregation, and cytokinesis. AURKB is also involved in the Survivin signaling pathway, where it interacts with Survivin, an inhibitor of apoptosis (IAP), to promote mitotic progression and inhibit apoptosis.^22^

 Furthermore, RRM2 plays a critical role in p53 signaling and pyrimidine metabolism, participating in DNA synthesis and repair. RRM2 functions within ribonucleotide reductase (RR) as a heterodimer with RRM1 or p53R2, ensuring nucleotide availability for DNA replication. Notably, mutant p53 enhances RRM2 expression, contributing to increased proliferation, chemoresistance, and poor prognosis in TNBC.^23^

 Binding site analysis identified Mitotic Arrest Deficient 2 Like 1 (MAD2L1) as a major target of hsa-miR-203a-3p, with seven predicted binding sites. MAD2L1 is a critical component of the spindle assembly checkpoint, ensuring accurate chromosome segregation during mitosis. Overexpression of MAD2L1 has been linked to chromosomal instability, aneuploidy, and tumor progression in multiple cancers, including TNBC.^24^ Functional assays demonstrated that exosomal delivery of hsa-miR-203a-3p (ExomiR) significantly downregulated MAD2L1, leading to reduced TNBC cell viability and proliferation. These findings correlate with MTT assay results, which showed that ExomiR-mediated inhibition of oncogenic targets suppressed TNBC viability through mechanisms involving cell senescence, cell cycle arrest, p53 signaling, and pyrimidine metabolism.

 Beyond its role in cell cycle regulation, hsa-miR-203a-3p was found to inhibit TNBC cell migration by targeting genes involved in Polycomb repressive complex signaling and phagosome formation, particularly Enhancer Of Zeste 2 Polycomb Repressive Complex 2 Subunit (EZH2), PHD Finger Protein 19 (PHF19), and Ras-Related Protein Rab-5A (RAB5A). EZH2, the catalytic subunit of Polycomb Repressive Complex 2 (PRC2), is a key epigenetic modifier that silences tumor suppressor genes, promoting aggressive TNBC phenotypes. PHF19, a regulatory component of PRC2, facilitates gene repression via pathways such as Wnt/β-catenin and TGF-β, both of which play crucial roles in epithelial-mesenchymal transition (EMT) and tumor invasiveness.^25,26^

 Moreover, RAB5A, a major regulator of endosomal trafficking, mediates the secretion of extracellular vesicles containing pro-metastatic factors, including matrix metalloproteinases. Overexpression of RAB5A promotes TNBC invasion and metastasis by facilitating the degradation of the extracellular matrix. Functional experiments demonstrated that hsa-miR-203a-3p-mediated inhibition of EZH2, PHF19, and RAB5A significantly impaired TNBC cell migration and invasion, highlighting its role as a metastasis-suppressive agent.^27^

 A limitation of this study is the absence of TEM imaging and Western blot analysis for exosomal surface markers such as CD63, CD81, and CD9. Due to facility constraints, exosome validation relied solely on NTA, which provided particle size distribution and phenotype. Future studies will incorporate multiple characterization methods to strengthen exosome identity confirmation. Although miRNA uptake was confirmed by qRT-PCR. This study identifies several high-confidence target genes through in silico prediction, direct experimental validation using Western blotting and luciferase reporter assays was not performed. These methods are essential for confirming the functional interaction between hsa-miR-203a-3p and its downstream targets. Future investigations will incorporate these assays to validate miRNA–mRNA binding and verify the regulatory effects at the protein level. Beside that, Although TNBC-specific transcriptomic data from GSE65194 were incorporated, integration with TCGA datasets was not performed in this study. Given the heterogeneity of breast cancer subtypes and challenges in subtype-specific annotation, TCGA-based validation will be an important focus of future investigations.

## Conclusion

 Delivery of hsa-miR-203a-3p using exosome-loaded systems was associated with a reduction in cell viability and migratory capacity of TNBC cells in vitro. Bioinformatic analyses suggest that hsa-miR-203a-3p may be involved in the regulation of genes related to cell cycle progression and migration; however, further experimental validation is required to confirm these regulatory interactions.

## Competing Interests

 All the authors declare that there are no conflicts of interest.

## Data Availability Statement

 Derived data supporting the findings of this study are available from the corresponding author on request.

## Ethical Approval

 Protocol of the present study was reviewed and approved by Ethical Committee of Faculty of Medicine, Public Health, and Nursing, Universitas Gadjah Mada, Yogyakarta, Indonesia (Approval number: KE/FK/1188/EC/2023).

## Supplementary File

Morphological observation of Hs578T cells.
