# Supplementary material for: Effect of Exosome-loaded hsa-miR-203a-3p on Triple Negative Breast Cancer Cell Viability, Migration, and Gene Targets: In Silico and In Vitro Study
Source: Adv Pharm Bull. 2026 May 23;16(2):352–62. doi: 10.34172/apb.45269 (PMC13745380; doi:10.34172/apb.45269)
Supplement: Supplementary file 1 [file apb-16-352-s001.pdf]

Adv Pharm Bull 2026;16(2):352-S1
doi:10.34172/apb.45269
https://apb.tbzmed.ac.ir

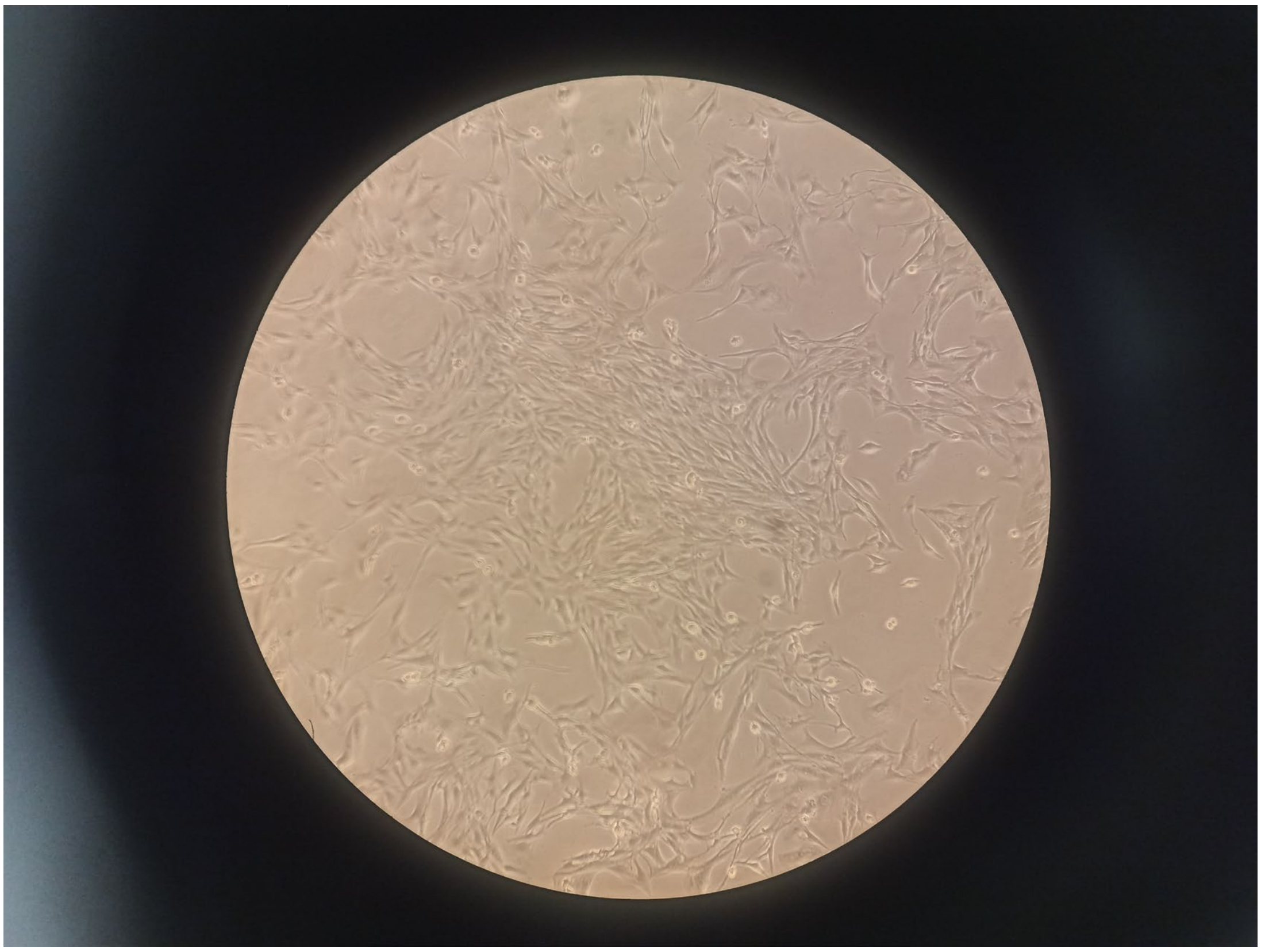

Figure S1. Hs578T cells control

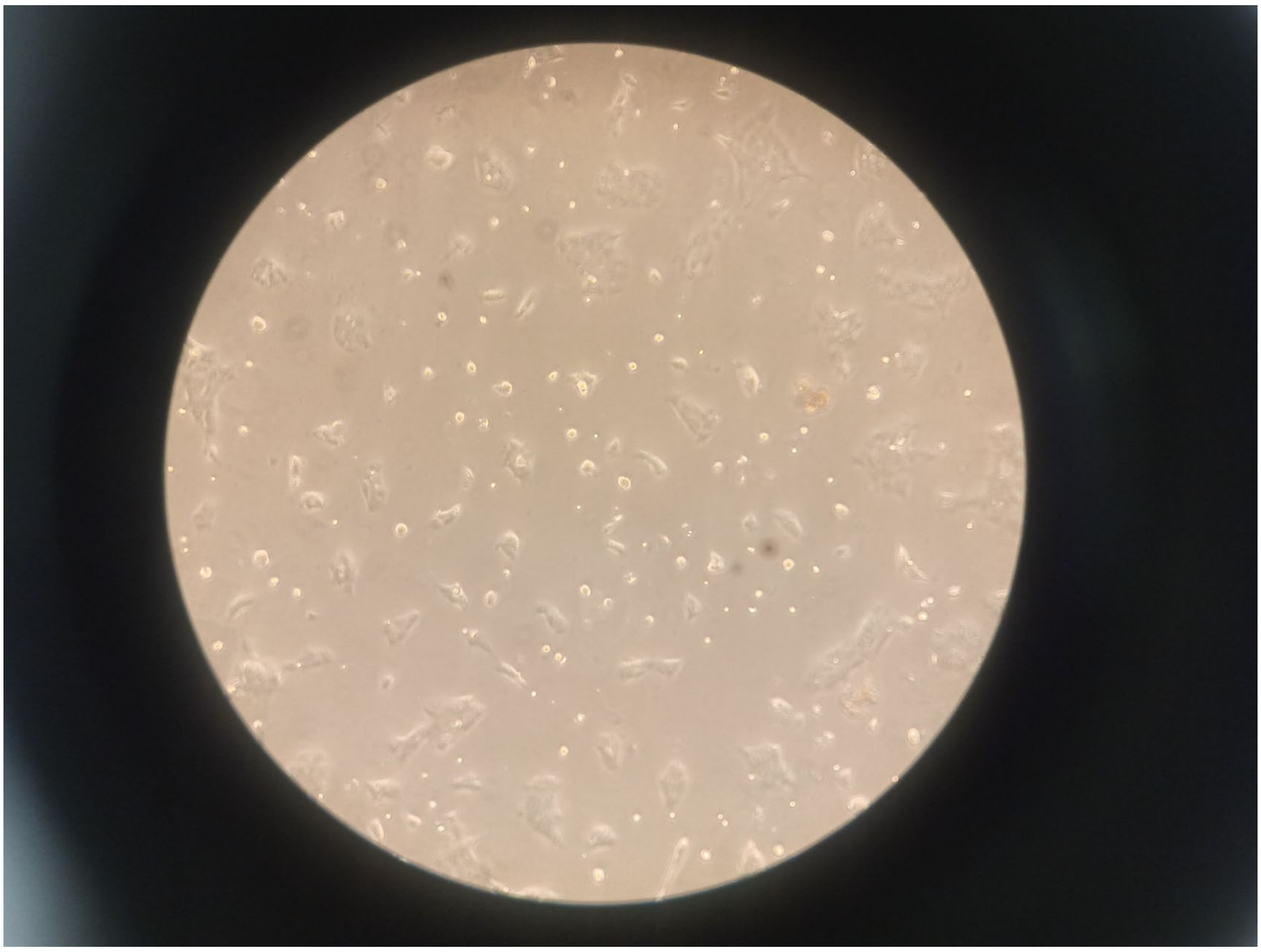

Figure S2. Hs578T cells Treatment ExomiR
